# An Updated Theoretical Framework for Vocal Hyperfunction

**DOI:** 10.1044/2020_AJSLP-20-00104

**Published:** 2020-10-02

**Authors:** Robert E. Hillman, Cara E. Stepp, Jarrad H. Van Stan, Matías Zañartu, Daryush D. Mehta

**Affiliations:** a Massachusetts General Hospital, Boston; b Harvard Medical School, Boston, MA; c MGH Institute of Health Professions, Boston, MA; d Boston University, MA; e Universidad Técnica Federico Santa María, Valparaíso, Chile

## Abstract

**Purpose:**

The purpose of this viewpoint article is to facilitate research on vocal hyperfunction (VH). VH is implicated in the most commonly occurring types of voice disorders, but there remains a pressing need to increase our understanding of the etiological and pathophysiological mechanisms associated with VH to improve the prevention, diagnosis, and treatment of VH-related disorders.

**Method:**

A comprehensive theoretical framework for VH is proposed based on an integration of prevailing clinical views and research evidence.

**Results:**

The fundamental structure of the current framework is based on a previous (simplified) version that was published over 30 years ago (Hillman et al., 1989). A central premise of the framework is that there are two primary manifestations of VH—phonotraumatic VH and nonphonotraumatic VH—and that multiple factors contribute and interact in different ways to cause and maintain these two types of VH. Key hypotheses are presented about the way different factors may contribute to phonotraumatic VH and nonphonotraumatic VH and how the associated disorders may respond to treatment.

**Conclusions:**

This updated and expanded framework is meant to help guide future research, particularly the design of longitudinal studies, which can lead to a refinement in knowledge about the etiology and pathophysiology of VH-related disorders. Such new knowledge should lead to further refinements in the framework and serve as a basis for improving the prevention and evidence-based clinical management of VH.

Vocal hyperfunction (VH) refers to “excessive perilaryngeal musculoskeletal activity” during phonation (Oates & Winkworth, 2008) and is considered an etiological component (Hillman et al., 1989) in the most frequently occurring types of voice disorders (Bhattacharyya, 2014). These include benign vocal fold lesions (e.g., nodules) and dysphonia that occurs in the absence of concurrent pathology (e.g., primary muscle tension dysphonia; Colton et al., 2006). The onset and chronic persistence of VH has been attributed to a variety of potential factors (see the VH framework in Figure 1), but the relative role of these factors in the etiology and chronic pathophysiology of VH remains uncertain. Although the general concept of VH is widely accepted and applied in clinical practice, effective prevention and clinical management of voice disorders associated with VH continue to be hampered by limited knowledge of underlying etiological and pathophysiological mechanisms.

**Figure 1.**
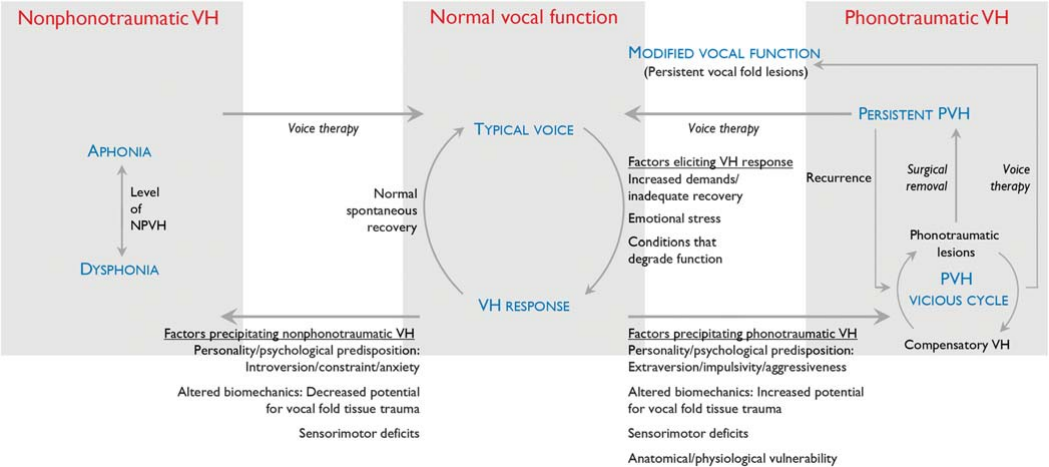
Theoretical framework for vocal hyperfunction (VH). The framework considers phonotraumatic VH (PVH) to be an etiological factor in “benign lesions of the lamina propria” (primarily vocal fold nodules, polyps, and reactive lesions) and possibly “non-intubation related vocal fold granuloma,” whereas nonphonotraumatic VH (NPVH) is considered to be an etiological factor in “primary muscle tension dysphonia.”

In this brief report, we propose a comprehensive theoretical framework for VH that is an updated and expanded version of a fundamental structure that was previously described over 30 years ago (Hillman et al., 1989). The updated framework is meant to assist VH research by organizing prevailing clinical perspectives and research evidence in a way that can help guide future investigations (including the generation of study designs and testable research hypotheses) and can be modified based on new findings. Such a framework for VH is also expected to be useful in clinical teaching and problem-solving.

## VH Framework

A schematic of the updated theoretical VH framework is shown in Figure 1. A central premise of the framework is that multiple factors contribute and interact in different ways to cause and maintain the different disorders linked to VH. The framework is based on an integration of some prevailing clinical views and research evidence. It illustrates key hypotheses about factors that may contribute to the two different manifestations of VH and how the associated conditions respond to treatment.

In the framework, different “states” of vocal function are shown in blue text. Labeled arrows indicate the directions of potential changes between functional states in the framework, along with hypotheses about what could contribute to those changes. All patients with VH may experience a variety of chronic voice-related symptoms that can include excessive activity of the perilaryngeal muscles during phonation (Oates & Winkworth, 2008), varying degrees of dysphonia and/or vocal limitations, vocal fatigue and/or increased vocal effort (Hunter et al., 2020; Solomon, 2008), and neck discomfort. The current framework maintains the fundamental structure of the earlier (simplified) version (Hillman et al., 1989), which postulated that there are two primary types of VH that are fundamentally different in terms of phonatory pathophysiology, that is, *adducted* VH versus *nonadducted* VH, which we now refer to more definitively as *phonotraumatic* VH (PVH—right side of the framework) and *nonphonotraumatic* VH (NPVH—left side of the framework). The change in terminology is due to (a) a realization that the original terminology (adducted vs. nonadducted) could cause confusion (e.g., clinical observations of adducted/compressed laryngeal structures with no trauma of vocal fold tissue), (b) the more straightforward reliance of the new terminology on the simple presence or absence of tissue trauma associated with VH, and (c) consistency with the wider adoption of the “phonotrauma” concept or terminology (Verdolini, 1999). In terms of the categories (Dimension 1. Categories of Conditions) in the *Classification Manual for Voice Disorders–I* (Verdolini et al., 2006), the framework considers PVH to be an etiological factor in “benign lesions of the lamina propria” (primarily vocal fold nodules, polyps, and reactive lesions) and possibly “non-intubation related vocal fold granuloma,” whereas NPVH is considered to be an etiological factor in “primary muscle tension dysphonia.”

The new framework expands upon the previous version by including implicit hypotheses about etiological factors associated with VH based on research that has been published since the original framework was proposed over 30 years ago (Hillman et al., 1989). The new framework now clearly differentiates between factors that can trigger an initial hyperfunctional vocal response that is typically temporary (center of the framework) and other factors that can subsequently precipitate the development of a chronic phonotraumatic or nonphonotraumatic hyperfunctional voice disorder. It also has more detailed hypotheses about pathological states, particularly with respect to the development of tissue trauma and the impact of treatment on the pathophysiology of phonotraumatic disorders.

### Normal Vocal Function

In the center section of the framework, a cycle is depicted going back and forth between typical voice production and a VH response (Fujiki et al., 2017). To use the terminology recently proposed by a group seeking consensus on the definitions of these terms (Hunter et al., 2020), the VH response can be considered the result of an increase in “vocal demand” that produces a concomitant increase in an individual’s “vocal demand response.” The vocal demand response can be experienced by the individual as increased “vocal effort” and/or “vocal fatigue.” According to Solomon (2008), vocal fatigue is often associated with the VH response as a precipitating and/or reactive factor. It is believed to be mediated by both physiological and psychological mechanisms (Hunter et al., 2020; Solomon, 2008) and is typically defined as “the self-report of an increased sense of effort with prolonged phonation” (Solomon, 2008). This cycle represents typical everyday scenarios that are experienced by most individuals with normal vocal function during their lifetime. In these situations, commonly occurring factors (“Factors eliciting VH response”) can interfere with normal voice production (primarily reducing vocal efficiency and/or causing vocal fatigue) and elicit a temporary VH response that ceases for most people when the eliciting condition resolves (vocal function returns to normal; Hunter & Titze, 2009; Whitling et al., 2015; Xue et al., 2019). As indicated, such factors can include (a) transient periods of increased *vocal demand* (causing an increased vocal demand response; Hunter et al., 2020) with inadequate vocal recovery time (e.g., prolonged loud talking that creates trauma-induced vocal fold edema, dysphonia, and/or vocal fatigue); (b) increased emotional stress triggering nonvolitional (autonomic nervous system [ANS]) heightened activity of the perilaryngeal muscles (Dietrich et al., 2012; Dietrich & Verdolini Abbott, 2012; Helou et al., 2018, 2013), making voice production less stable and more effortful or hyperfunctional; and (c) conditions such as an upper respiratory infection, laryngopharyngeal reflux (J. T. Cohen et al., 2002; Koufman, 1991; Koufman et al., 2000), environmental irritants (e.g., smoke and chemical fumes), dehydration (Verdolini et al., 2002; Verdolini-Marston et al., 1990), and so forth, that typically degrade the vibratory capability of the vocal fold mucosa and make phonation more effortful or hyperfunctional.

### Factors Precipitating PVH and NPVH

The rest of the VH framework is devoted to hypotheses underlying the etiology of the two main types of chronic VH. We postulate that, in a minority of individuals (those who develop hyperfunctional voice disorders), once the hyperfunctional response is triggered, it does not resolve but becomes chronic, even in cases in which the original factors that elicited VH may no longer be present. We further hypothesize that there are differences among individuals in terms of some key factors (and interactions among key factors) that largely determine whether they develop PVH or NPVH, which are listed below the two opposing arrows that radiate out from the normative VH response and point toward PVH or NPVH.

### Personality/Psychological Predisposition

Numerous studies have identified personality traits that differentiate between healthy controls and patients with PVH or NPVH (McHugh-Munier et al., 1997; Ng et al., 2013; Roy et al., 2000a, 2000b; van Mersbergen et al., 2008). In general, these studies support the *trait theory* of voice disorders (Roy & Bless, 2000), which characterizes PVH patients as extroverted, impulsive, and potentially vocally aggressive or dominant, whereas patients with NPVH are described as introverted with a tendency to be behaviorally constrained and anxious. The prevailing view is that the personality factors associated with PVH could predispose those patients to engage in detrimental vocal behaviors (e.g., loud talking), whereas the personality factors associated with NPVH could predispose those patients to experience higher-than-normal levels of psychological stress in speaking situations, which could, in turn, result in stress-induced hyperactivity (or imbalanced activity) of the perilaryngeal muscles, thus contributing to their vocal difficulties. The hypothesized stress-reactive response for NPVH patients gains further support from studies showing evidence of the link between stressor-induced reactivity of the ANS and increased activity or overactivity of the perilaryngeal (Dietrich et al., 2012; Dietrich & Verdolini Abbott, 2012) and intrinsic laryngeal (Helou et al., 2018, 2013) muscles. There is also initial evidence of a potential link between ANS dysfunction and NPVH (Demmink-Geertman & Dejonckere, 2002; Farah Paes et al., 2014; Park & Behlau, 2011).

### Altered Biomechanics

Several studies have provided evidence that the pathophysiological mechanisms (biomechanics) associated with the two forms of VH are quite different. Glottal aerodynamic studies have shown that patients with PVH need to produce abnormally high values for subglottal air pressure, modulated airflow (reflecting increased amplitude of vocal fold vibration), and maximum flow declination rate (reflecting increased vocal fold closure velocity and collision forces) to attain normal vocal sound pressure levels (Espinoza, Zañartu, et al., 2017; Hillman et al., 1989, 1990). These results reflect increased potential to cause trauma to vocal fold tissue, thus contributing to the chronic presence of vocal fold lesions and associated dysphonia. Conversely, patients with NPVH display abnormal phonatory function that is inefficient, with increases in subglottal air pressure and open quotient (reflecting incomplete glottal closure) without increased modulated airflow and maximum flow declination rate; this can produce dysphonia but has decreased potential to cause trauma to vocal fold tissue (Espinoza, Zañartu, et al., 2017; Hillman et al., 1989). In addition, recent results using ambulatory voice monitoring further corroborate previous descriptions of the mechanisms associated with PVH by showing that the daily vocal function of these patients can be differentiated from healthy controls based on the long-term distributional characteristics of two parameters (combined in a logistic regression) that indicate a higher magnitude of phonatory forces and the tendency to more frequently employ more abrupt glottal closure (Van Stan et al., 2020). Measures of relative fundamental frequency (shown to be sensitive to VH) have also provided additional indirect evidence of the potential difference in pathophysiological mechanisms between PVH and NPVH (Heller Murray et al., 2017).

The PVH section of the framework includes what clinicians often describe as the “vicious cycle” (Hillman et al., 1989), which assumes that a major contributor to the chronic nature and worsening of this condition is a progressive increase in hyperfunction (which increases the forces causing vocal fold trauma) to maintain an adequate level of phonation (the state referred to in the framework as “Compensatory VH”) as trauma-inducing damage to vocal fold tissue increases. Support for this aspect of PVH pathogenesis has been provided by computational modeling (Galindo et al., 2017; Zañartu et al., 2014), where it was shown that compensating (e.g., with increased subglottal pressure) for a VH-onset condition (e.g., persistent posterior glottal gap extending into the membranous glottis) can restore an acoustic target (e.g., sound pressure level), but it also yields high vocal fold collision forces. These modeling efforts also show that the risk of developing phonotrauma with these increased collision forces is reflected in elevated aerodynamic measures that are in agreement with laboratory (Espinoza, Mehta, et al., 2017; Hillman et al., 1989, 1990) and ambulatory studies (Cortés et al., 2018; Van Stan et al., 2020). The NPVH section of the framework shows the hypothesized relationship between the level or degree of VH and its impact on voice production, with the severity of dysphonia increasing as the level of VH increases until aphonia occurs (Hillman et al., 1989).

### Sensorimotor Deficits

Recent studies have begun to show evidence of potential sensorimotor deficits in patients with VH that could contribute to the development of the associated chronic voice disorders. Studies using perturbations in the fundamental frequency of auditory feedback have demonstrated that some individuals diagnosed with hyperfunctional voice disorders (both PVH and NPVH) produce nontypical vocal responses, suggesting atypical sensorimotor control (Stepp et al., 2017; Ziethe et al., 2019). These results are supported by other works suggesting that auditory acuity (Tam et al., 2018) and auditory–motor targets (McKenna et al., 2020) differ in individuals with VH relative to controls. There is also preliminary evidence from neural imaging (Kryshtopava et al., 2017) and transcranial magnetic stimulation (Samargia et al., 2016) studies of differences in sensorimotor control mechanisms between patients with NPVH and controls that could conceivably play a role in the types of maladaptive laryngeal phonatory function manifest in patients with NPVH. However, it is not yet clear whether these sensorimotor deficits are etiological or compensatory (Naunheim et al., 2019).

### Anatomical/Physiological Vulnerability

For PVH, the framework also includes the hypothesis that congenital/inherent anatomical and/or physiological characteristics may make some individuals more susceptible to vocal fold tissue damage. This is a common notion that is based on widely shared clinical impressions but has yet to be empirically verified (Mi et al., 2010). It is hoped that an outcome of future research will be more specific phenotyping of patients with hyperfunctional voice disorders and that this new evidence could provide a more solid basis for systematically investigating the potential impact of such additional factors that precipitate PVH.

### PVH Treatment

The various treatment options for phonotraumatic lesions are represented in the PVH section of the VH framework in Figure 1 (italicized text), including surgical removal, voice therapy, or a combination of both. The hypothesized outcome for surgical removal of nodules (without voice therapy afterward) reflects the prevailing clinical view that phonotraumatic lesions will recur (following unresolved, persistent PVH) without therapeutic “retraining” of the voice; however, aside from clinical anecdotes, there is a lack of empirical evidence that recurrence rates are related to whether or not patients receive postsurgical voice therapy (Colton et al., 2006; Kunduk & McWhorter, 2009; Zeitels et al., 2002). The outcome shown for using voice therapy as the sole treatment (“Modified vocal function”) is based on our clinical and research experience (Holmberg et al., 2003, 2001), where we have observed that patients who are satisfied with their vocal function following an initial course of voice therapy (and elect not to have surgery) still have nodules when carefully examined with high-quality videostroboscopy. This is somewhat at odds with the long-standing clinical view that nodules can often resolve with effective voice therapy (S. M. Cohen & Garrett, 2007; Holbrook et al., 1974; Leonard, 2009). However, there is a paucity of long-term outcome data for patients with phonotraumatic lesions who receive only voice therapy. Thus, the status for such patients is described in the framework as “Modified vocal function” and is purposely positioned between the PVH and Normal vocal function blocks of the framework. Only the treatment path that includes surgery followed by voice therapy indicates that a return to Normal vocal function is possible.

### NPVH Treatment

The NPVH section of the framework contains less detail than the PVH section because there is no known mechanism comparable to the assumed major role of the “vicious cycle” in PVH and because there is only one standard treatment option (voice therapy). This lack of detail also reflects the fact that, in general, relatively less is known about the etiological and pathophysiological mechanisms that are responsible for NPVH, including whether or not there are subgroups of patients within this broad classification, as has been suggested by some authors based on endoscopic observations (Morrison et al., 1986) or symptomatic presentation (Spencer, 2015). NPVH is typically viewed as the persistence of a hyperfunctional laryngeal response to some condition that impacts voice production (see “Factors eliciting VH response” in the Normal vocal function block of the framework); however, it is not known why such patients literally seem to “get stuck” (or habituate) using these maladaptive vocal behaviors. It is hoped that emerging evidence from sensorimotor and neural imaging/stimulation studies of NPVH patients (see the previous Sensorimotor Deficits section) will eventually help better elucidate potential etiological mechanisms. The framework also portrays the outcome of (successful) voice therapy for NPVH as restoring normal vocal function. However, much of the evidence to support the restoration of normal phonation is based mostly on patients’ self-ratings of vocal function (Ramig & Verdolini, 1998; Ruotsalainen et al., 2008; Speyer, 2008). There is a lack of information about the extent to which self-reported improvements in vocal function are due to changes in underlying phonatory mechanisms and/or modifications in other factors, including the type and extent of voice use, and what impact such variations in outcomes may have on long-term efficacy.

## Need for Longitudinal Research to Assess and Refine the Framework

Assessing the broad range of factors, interactions among factors, concepts, and hypotheses that comprise the VH framework will continue to require a concerted interdisciplinary effort. A major challenge in better elucidating the etiology and pathophysiology of VH-related disorders is that it is difficult to determine to what extent observed abnormalities in vocal function or other measures (e.g., psychosocial) represent causative and/or reactive/compensatory mechanisms once the disorders are present. Monitoring groups of individuals with normal vocal function (possibly focusing on high-risk populations) that are large enough to possibly detect the onset of hyperfunctional voice disorders in a subgroup has not been accomplished, nor is it currently feasible. A potential alternative solution that is more practical involves the longitudinal (repeated) assessment of patients starting with pretreatment and then, after each phase of treatment (surgery, voice therapy, etc.), including long-term follow-up (e.g., 6 months after final treatment). Measures that not only migrate toward normal/typical ranges posttreatment but also correlate with other short- and long-term outcomes are expected to be critical to providing insight into etiological and/or primary pathophysiological mechanisms of VH.

## References

[bib1] Bhattacharyya, N. (2014). The prevalence of voice problems among adults in the United States. The Laryngoscope, 124(10), 2359–2362. https://doi.org/10.1002/lary.24740 24782443 10.1002/lary.24740

[bib2] Cohen, J. T. , Bach, K. K. , Postma, G. N. , & Koufman, J. A. (2002). Clinical manifestations of laryngopharyngeal reflux. Ear, Nose & Throat Journal, 81(9, Suppl. 2), 19–23. https://www.ncbi.nlm.nih.gov/pubmed/12353427 12353427

[bib3] Cohen, S. M. , & Garrett, C. G. (2007). Utility of voice therapy in the management of vocal fold polyps and cysts. Otolaryngology—Head & Neck Surgery, 136(5), 742–746. https://doi.org/10.1016/j.otohns.2006.12.009 17478208 10.1016/j.otohns.2006.12.009

[bib4] Colton, R. H. , Casper, J. K. , & Leonard, R. J. (2006). Understanding voice problems: A physiological perspective for diagnosis and treatment. Lippincott Williams & Wilkins.

[bib5] Cortés, J. P. , Espinoza, V. M. , Ghassemi, M. , Mehta, D. D. , Van Stan, J. H. , Hillman, R. E. , Guttag, J. V. , & Zañartu, M. (2018). Ambulatory assessment of phonotraumatic vocal hyperfunction using glottal airflow measures estimated from neck-surface acceleration. PLOS ONE, 13(12), Article e0209017. https://doi.org/10.1371/journal.pone.0209017 10.1371/journal.pone.0209017 PMC6301575 30571719

[bib6] Demmink-Geertman, L. , & Dejonckere, P. H. (2002). Nonorganic habitual dysphonia and autonomic dysfunction. Journal of Voice, 16(4), 549–559. https://doi.org/10.1016/s0892-1997(02)00130-3 12512642 10.1016/s0892-1997(02)00130-3

[bib7] Dietrich, M. , Andreatta, R. D. , Jiang, Y. , Joshi, A. , & Stemple, J. C. (2012). Preliminary findings on the relation between the personality trait of stress reaction and the central neural control of human vocalization. International Journal of Speech-Language Pathology, 14(4), 377–389. https://doi.org/10.3109/17549507.2012.688865 22698155 10.3109/17549507.2012.688865

[bib8] Dietrich, M. , & Verdolini Abbott, K. (2012). Vocal function in introverts and extraverts during a psychological stress reactivity protocol. Journal of Speech, Language, and Hearing Research, 55(3), 973–987. https://doi.org/10.1044/1092-4388(2011/10-0344) 10.1044/1092-4388(2011/10-0344) 22232397

[bib9] Espinoza, V. M. , Mehta, D. D. , Van Stan, J. H. , Hillman, R. E. , & Zañartu, M. (2017). Uncertainty of glottal airflow estimation during continuous speech using impedance-based inverse filtering of the neck-surface acceleration signal. The Journal of the Acoustical Society of America, 141, 3579. https://doi.org/10.1121/1.4987622

[bib10] Espinoza, V. M. , Zañartu, M. , Van Stan, J. H. , Mehta, D. D. , & Hillman, R. E. (2017). Glottal aerodynamic measures in women with phonotraumatic and nonphonotraumatic vocal hyperfunction. Journal of Speech, Language, and Hearing Research, 60(8), 2159–2169. https://doi.org/10.1044/2017_JSLHR-S-16-0337 10.1044/2017_JSLHR-S-16-0337 PMC5829799 28785762

[bib11] Farah Paes, C. , Copelli Zambon, F. , & Behlau, M. (2014). Signs and symptoms of the autonomic dysfunction in teachers. Revista CEFAC, 16(3), 957–966. https://doi.org/10.1590/1982-021620141613

[bib12] Fujiki, R. B. , Chapleau, A. , Sundarrajan, A. , McKenna, V. , & Sivasankar, M. P. (2017). The interaction of surface hydration and vocal loading on voice measures. Journal of Voice, 31(2), 211–217. https://doi.org/10.1016/j.jvoice.2016.07.005 27522343 10.1016/j.jvoice.2016.07.005

[bib13] Galindo, G. E. , Peterson, S. D. , Erath, B. D. , Castro, C. , Hillman, R. E. , & Zañartu, M. (2017). Modeling the pathophysiology of phonotraumatic vocal hyperfunction with a triangular glottal model of the vocal folds. Journal of Speech, Language, and Hearing Research, 60(9), 2451–2471. https://doi.org/10.1044/2017_JSLHR-S-16-0412 10.1044/2017_JSLHR-S-16-0412 PMC5831616 28837719

[bib14] Heller Murray, E. S. , Lien, Y. S. , Van Stan, J. H. , Mehta, D. D. , Hillman, R. E. , Pieter Noordzij, J. , & Stepp, C. E. (2017). Relative fundamental frequency distinguishes between phonotraumatic and non-phonotraumatic vocal hyperfunction. Journal of Speech, Language, and Hearing Research, 60(6), 1507–1515. https://doi.org/10.1044/2016_JSLHR-S-16-0262 10.1044/2016_JSLHR-S-16-0262 PMC5544410 28595317

[bib15] Helou, L. B. , Rosen, C. A. , Wang, W. , & Verdolini Abbott, K. (2018). Intrinsic laryngeal muscle response to a public speech preparation stressor. Journal of Speech, Language, and Hearing Research, 61(7), 1525–1543. https://doi.org/10.1044/2018_JSLHR-S-17-0153 10.1044/2018_JSLHR-S-17-0153 PMC6195061 29922837

[bib16] Helou, L. B. , Wang, W. , Ashmore, R. C. , Rosen, C. A. , & Verdolini Abbott, K. (2013). Intrinsic laryngeal muscle activity in response to autonomic nervous system activation. The Laryngoscope, 123(11), 2756–2765. https://doi.org/10.1002/lary.24109 23620064 10.1002/lary.24109

[bib17] Hillman, R. E. , Holmberg, E. B. , Perkell, J. S. , Walsh, M. , & Vaughan, C. (1989). Objective assessment of vocal hyperfunction: An experimental framework and initial results. Journal of Speech and Hearing Research, 32(2), 373–392. https://doi.org/10.1044/jshr.3202.373 2739390 10.1044/jshr.3202.373

[bib18] Hillman, R. E. , Holmberg, E. B. , Perkell, J. S. , Walsh, M. , & Vaughan, C. (1990). Phonatory function associated with hyperfunctionally related vocal fold lesions. Journal of Voice, 4(1), 52–63. https://doi.org/10.1016/S0892-1997(05)80082-7

[bib19] Holbrook, A. , Rolnick, M. I. , & Bailey, C. W. (1974). Treatment of vocal abuse disorders using a vocal intensity controller. Journal of Speech and Hearing Disorders, 39(3), 298–303. https://doi.org/10.1044/jshd.3903.298 10.1044/jshd.3903.298 4416734

[bib20] Holmberg, E. B. , Doyle, P. , Perkell, J. S. , Hammarberg, B. , & Hillman, R. E. (2003). Aerodynamic and acoustic voice measurements of patients with vocal nodules: Variation in baseline and changes across voice therapy. Journal of Voice, 17(3), 269–282. https://doi.org/10.1067/S0892-1997(03)00076-6 14513951 10.1067/s0892-1997(03)00076-6

[bib21] Holmberg, E. B. , Hillman, R. E. , Hammarberg, B. , Södersten, M. , & Doyle, P. (2001). Efficacy of a behaviorally based voice therapy protocol for vocal nodules. Journal of Voice, 15(3), 395–412. https://doi.org/10.1016/S0892-1997(01)00041-8 11575636 10.1016/S0892-1997(01)00041-8

[bib22] Hunter, E. J. , Cantor-Cutiva, L. C. , van Leer, E. , van Mersbergen, M. , Nanjundeswaran, C. D. , Bottalico, P. , Sandage, M. J. , & Whitling, S. (2020). Toward a consensus description of vocal effort, vocal load, vocal loading, and vocal fatigue. Journal of Speech, Language, and Hearing Research, 63(2), 509–532. https://doi.org/10.1044/2019_JSLHR-19-00057 10.1044/2019_JSLHR-19-00057 PMC7210446 32078404

[bib23] Hunter, E. J. , & Titze, I. R. (2009). Quantifying vocal fatigue recovery: Dynamic vocal recovery trajectories after a vocal loading exercise. Annals of Otology, Rhinology & Laryngology, 118(6), 449–460. https://doi.org/10.1177/000348940911800608 10.1177/000348940911800608 PMC3311979 19663377

[bib24] Koufman, J. A. (1991). The otolaryngologic manifestations of gastroesophageal reflux disease (GERD): A clinical investigation of 225 patients using ambulatory 24-hour pH monitoring and an experimental investigation of the role of acid and pepsin in the development of laryngeal injury. The Laryngoscope, 101(S53), 1–78. https://doi.org/10.1002/lary.1991.101.s53.1 10.1002/lary.1991.101.s53.1 1895864

[bib25] Koufman, J. A. , Amin, M. R. , & Panetti, M. (2000). Prevalence of reflux in 113 consecutive patients with laryngeal and voice disorders. Otolaryngology—Head & Neck Surgery, 123(4), 385–388. https://doi.org/10.1067/mhn.2000.109935 11020172 10.1067/mhn.2000.109935

[bib26] Kryshtopava, M. , Van Lierde, K. , Meerschman, I. , D’Haeseleer, E. , Vandemaele, P. , Vingerhoets, G. , & Claeys, S. (2017). Brain activity during phonation in women with muscle tension dysphonia: An fMRI study. Journal of Voice, 31(6), 675–690. https://doi.org/10.1016/j.jvoice.2017.03.010 28410773 10.1016/j.jvoice.2017.03.010

[bib27] Kunduk, M. , & McWhorter, A. J. (2009). True vocal fold nodules: The role of differential diagnosis. Current Opinion in Otolaryngology & Head and Neck Surgery, 17(6), 449–452. https://doi.org/10.1097/MOO.0b013e3283328b6d 19779347 10.1097/MOO.0b013e3283328b6d

[bib28] Leonard, R. (2009). Voice therapy and vocal nodules in adults. Current Opinion in Otolaryngology & Head and Neck Surgery, 17(6), 453–457. https://doi.org/10.1097/MOO.0b013e3283317fd2 19741535 10.1097/MOO.0b013e3283317fd2

[bib29] McHugh-Munier, C. , Scherer, K. R. , Lehmann, W. , & Scherer, U. (1997). Coping strategies, personality, and voice quality in patients with vocal fold nodules and polyps. Journal of Voice, 11(4), 452–461. https://doi.org/10.1016/s0892-1997(97)80042-2 9422280 10.1016/s0892-1997(97)80042-2

[bib30] McKenna, V. S. , Hylkema, J. A. , Tardif, M. C. , & Stepp, C. E. (2020). Voice onset time in individuals with hyperfunctional voice disorders: Evidence for disordered vocal motor control. Journal of Speech, Language, and Hearing Research, 63(2), 405–420. https://doi.org/10.1044/2019_JSLHR-19-00135 10.1044/2019_JSLHR-19-00135 PMC7210440 32013664

[bib31] Mi, Q. , Li, N. Y.-K. , Ziraldo, C. , Ghuma, A. , Mikheev, M. , Squires, R. , Okonkwo, D. O. , Verdolini-Abbott, K. , Constantine, G. , & An, G. (2010). Translational systems biology of inflammation: Potential applications to personalized medicine. Personalized Medicine, 7(5), 549–559. https://doi.org/10.2217/pme.10.45 21339856 10.2217/pme.10.45 PMC3041597

[bib32] Morrison, M. D. , Nichol, H. , & Rammage, L. A. (1986). Diagnostic criteria in functional dysphonia. The Laryngoscope, 96(1), 1–8. https://doi.org/10.1288/00005537-198601000-00001 10.1288/00005537-198601000-00001 3941573

[bib33] Naunheim, M. L. , Yung, K. C. , Schneider, S. L. , Henderson-Sabes, J. , Kothare, H. , Mizuiri, D. , Klein, D. J. , Houde, J. F. , Nagarajan, S. S. , & Cheung, S. W. (2019). Vocal motor control and central auditory impairments in unilateral vocal fold paralysis. The Laryngoscope, 129(9), 2112–2117. https://doi.org/10.1002/lary.27680 30484858 10.1002/lary.27680

[bib34] Ng, J. H. , Lo, S. , Lim, F. , Goh, S. , & Kanagalingam, J. (2013). Association between anxiety, Type A personality, and treatment outcome of dysphonia due to benign causes. Otolaryngology—Head & Neck Surgery, 148(1), 96–102. https://doi.org/10.1177/0194599812465592 23112274 10.1177/0194599812465592

[bib35] Oates, J. , & Winkworth, A. (2008). Current knowledge, controversies and future directions in hyperfunctional voice disorders. International Journal of Speech-Language Pathology, 10(4), 267–277. https://doi.org/10.1080/17549500802140153 20840042 10.1080/17549500802140153

[bib36] Park, K. , & Behlau, M. (2011). Signs and symptoms of autonomic dysfunction in dysphonic individuals. Jornal da Sociedade Brasileira de Fonoaudiologia, 23(2), 164–169. https://doi.org/10.1590/s2179-64912011000200014 21829933 10.1590/s2179-64912011000200014

[bib37] Ramig, L. O. , & Verdolini, K. (1998). Treatment efficacy: Voice disorders. Journal of Speech, Language, and Hearing Research, 41(1), S101–S116. https://doi.org/10.1044/jslhr.4101.s101 10.1044/jslhr.4101.s101 9493749

[bib38] Roy, N. , & Bless, D. M. (2000). Personality traits and psychological factors in voice pathology: A foundation for future research. Journal of Speech, Language, and Hearing Research, 43(3), 737–748. https://doi.org/10.1044/jslhr.4303.737 10.1044/jslhr.4303.737 10877442

[bib39] Roy, N. , Bless, D. M. , & Heisey, D. (2000a). Personality and voice disorders: A multitrait-multidisorder analysis. Journal of Voice, 14(4), 521–548. https://doi.org/10.1016/S0892-1997(00)80009-0 11130110 10.1016/s0892-1997(00)80009-0

[bib40] Roy, N. , Bless, D. M. , & Heisey, D. (2000b). Personality and voice disorders: A superfactor trait analysis. Journal of Speech, Language, and Hearing Research, 43(3), 749–768. https://doi.org/10.1044/jslhr.4303.749 10.1044/jslhr.4303.749 10877443

[bib41] Ruotsalainen, J. , Sellman, J. , Lehto, L. , & Verbeek, J. (2008). Systematic review of the treatment of functional dysphonia and prevention of voice disorders. Otolaryngology—Head & Neck Surgery, 138(5), 557–565. https://doi.org/10.1016/j.otohns.2008.01.014 18439458 10.1016/j.otohns.2008.01.014

[bib42] Samargia, S. , Schmidt, R. , & Kimberley, T. J. (2016). Cortical silent period reveals differences between adductor spasmodic dysphonia and muscle tension dysphonia. Neurorehabilitation & Neural Repair, 30(3), 221–232. https://doi.org/10.1177/1545968315591705 26089309 10.1177/1545968315591705 PMC4684481

[bib43] Solomon, N. P. (2008). Vocal fatigue and its relation to vocal hyperfunction. International Journal of Speech-Language Pathology, 10(4), 254–266. https://doi.org/10.1080/14417040701730990 20840041 10.1080/14417040701730990

[bib44] Spencer, M. L. (2015). Muscle tension dysphonia: A rationale for symptomatic subtypes, expedited treatment, and increased therapy compliance. SIG 3 Perspectives on Voice and Voice Disorders, 25(1), 5–15. https://doi.org/10.1044/vvd25.1.5

[bib45] Speyer, R. (2008). Effects of voice therapy: A systematic review. Journal of Voice, 22(5), 565–580. https://doi.org/10.1016/j.jvoice.2006.10.005 17509828 10.1016/j.jvoice.2006.10.005

[bib46] Stepp, C. E. , Lester-Smith, R. A. , Abur, D. , Daliri, A. , Pieter Noordzij, J. , & Lupiani, A. A. (2017). Evidence for auditory-motor impairment in individuals with hyperfunctional voice disorders. Journal of Speech, Language, and Hearing Research, 60(6), 1545–1550. https://doi.org/10.1044/2017_JSLHR-S-16-0282 10.1044/2017_JSLHR-S-16-0282 PMC5544411 28590007

[bib47] Tam, K. H. , Carding, P. , Heard, R. , & Madhill, C. J. (2018). The relationship between voice quality and pitch discrimination ability in a population with features of mild vocal hyperfunction [Paper presentation] . Voice Foundation’s 47th Annual Symposium, Philadelphia, PA, United States.

[bib48] van Mersbergen, M. , Patrick, C. , & Glaze, L. (2008). Functional dysphonia during mental imagery: Testing the trait theory of voice disorders. Journal of Speech, Language, and Hearing Research, 51(6), 1405–1423. https://doi.org/10.1044/1092-4388(2008/06-0216) 10.1044/1092-4388(2008/06-0216) 18664709

[bib49] Van Stan, J. H. , Mehta, D. D. , Ortiz, A. J. , Burns, J. A. , Toles, L. E. , Marks, K. L. , Vangel, M. , Hron, T. , Zeitels, S. , & Hillman, R. E. (2020). Differences in weeklong ambulatory vocal behavior between female patients with phonotraumatic lesions and matched controls. Journal of Speech, Language, and Hearing Research, 63(2), 372–384. https://doi.org/10.1044/2019_JSLHR-19-00065 10.1044/2019_JSLHR-19-00065 PMC7210443 31995428

[bib50] Verdolini, K. (1999). Critical analysis of common terminology in voice therapy. Phonoscope, 2(1), 1–8.

[bib51] Verdolini, K. , Min, Y. , Titze, I. R. , Lemke, J. , Brown, K. , van Mersbergen, M. , Jiang, J. , & Fisher, K. (2002). Biological mechanisms underlying voice changes due to dehydration. Journal of Speech, Language, and Hearing Research, 45(2), 268–281. https://doi.org/10.1044/1092-4388(2002/021) 10.1044/1092-4388(2002/021) 12003510

[bib52] Verdolini, K. , Rosen, C. , & Branski, R. C. (Eds.). (2006). Classification manual for voice disorders–I, Special Interest Division 3, Voice and Voice Disorders, American Speech-Language-Hearing Division. Erlbaum.

[bib53] Verdolini-Marston, K. , Titze, I. R. , & Druker, D. G. (1990). Changes in phonation threshold pressure with induced conditions of hydration. Journal of Voice, 4(2), 142–151. https://doi.org/10.1016/S0892-1997(05)80139-0

[bib54] Whitling, S. , Rydell, R. , & Lyberg Ahlander, V. (2015). Design of a clinical vocal loading test with long-time measurement of voice. Journal of Voice, 29(2), 261.E13–261.E27. https://doi.org/10.1016/j.jvoice.2014.07.012 10.1016/j.jvoice.2014.07.012 25499518

[bib55] Xue, C. , Kang, J. , Hedberg, C. , Zhang, Y. , & Jiang, J. J. (2019). Dynamically monitoring vocal fatigue and recovery using aerodynamic, acoustic, and subjective self-rating measurements. Journal of Voice, 33(5), 809.E11–809.E18. https://doi.org/10.1016/j.jvoice.2018.03.014 10.1016/j.jvoice.2018.03.014 31543208

[bib56] Zañartu, M. , Galindo, G. E. , Erath, B. D. , Peterson, S. D. , Wodicka, G. R. , & Hillman, R. E. (2014). Modeling the effects of a posterior glottal opening on vocal fold dynamics with implications for vocal hyperfunction. The Journal of the Acoustical Society of America, 136(6), 3262–3271. https://doi.org/10.1121/1.4901714 25480072 10.1121/1.4901714 PMC4257958

[bib57] Zeitels, S. M. , Hillman, R. E. , Desloge, R. , Mauri, M. , & Doyle, P. (2002). Phonomicrosurgery in singers and performing artists: Treatment outcomes, management theories, and future directions. Annals of Otology, Rhinology & Laryngology, 111(Suppl. 12), 21–40. https://doi.org/10.1177/0003489402111S1203 10.1177/0003489402111s1203 12498380

[bib58] Ziethe, A. , Petermann, S. , Hoppe, U. , Greiner, N. , Bruning, M. , Bohr, C. , & Dollinger, M. (2019). Control of fundamental frequency in dysphonic patients during phonation and speech. Journal of Voice, 33(6), 851–859. https://doi.org/10.1016/j.jvoice.2018.07.001 30143332 10.1016/j.jvoice.2018.07.001

